# Diverged morphology changes of astrocytic and neuronal primary cilia under reactive insults

**DOI:** 10.1186/s13041-020-00571-y

**Published:** 2020-03-02

**Authors:** Ashley Sterpka, Juan Yang, Matthew Strobel, Yuxin Zhou, Connor Pauplis, Xuanmao Chen

**Affiliations:** Department of Molecular, Cellular and Biomedical Sciences, College of Life Sciences and Agriculture, University of New Hampshire, 389 Rudman Hall, 46 College Road, Durham, NH 03824 USA

**Keywords:** Primary cilia, Astrocytes, Arl13b, AC3, Traumatic brain injury, Epilepsy

## Abstract

Primary cilia are centriole-derived sensory organelles that are present in most mammalian cells, including astrocytes and neurons. Evidence is emerging that astrocyte and neuronal primary cilia demonstrate a dichotomy in the mature mouse brain. However, it is unknown how astrocytic and neuronal primary cilia change their morphology and ciliary proteins when exposed to reactive insults including epilepsy and traumatic brain injury. We used a double transgenic mouse strain (Arl13b-mCherry; Centrin2-GFP), in which we found spontaneous seizures, and a cortical injury model to examine the morphological changes of astrocytic and neuronal primary cilia under reactive conditions. Transgenic overexpression of Arl13b drastically increases the length of astrocytic and neuronal primary cilia in the hippocampus, as well as the cilia lengths of cultured astrocytes and neurons. Spontaneous seizures shorten Arl13b-positive astrocytic cilia and AC3-positive neuronal cilia in the hippocampus. In a cortical injury model, Arl13b is not detectable in primary cilia, but Arl13b protein relocates to the cell body and has robust expression in the proximity of injured tissues. In contrast, the number of AC3-positive cilia near injured tissues remains unchanged, but their lengths become shorter. These results on astrocytic cilia implicate Arl13b in regulating astrocyte proliferation and tissue regeneration, while the shortening of AC3-positive cilia suggests adaptive changes of neuronal primary cilia under excitotoxicity.

## Introduction

Primary cilia are microtubule-based sensory organelles present in most mammalian cells, including neurons and astrocytes in the brain [1, 2]. The microenvironment of primary cilia is distinct from actin-based microdomains such as microvilli and synapses. During the G0 phase, the primary cilium emanates from the basal body, which is a special form of the mother centriole [3]. In quiescent cells, primary cilia are exquisitely sensitive to extracellular signals, serving as a hub to integrate signals to modulate a variety of cellular functions including neuronal activity [2, 4, 5]. In dividing cells, primary cilia engage directly with the cell cycle, and dismantling primary cilia is a prerequisite for the release of centrioles and formation of the mitotic spindle [6]. Thus, primary cilia have two diverged classes of function: to serve as the “cell antenna” [2, 7] to detect extracellular signals and to function as the “keeper of the key for cell division” [3] to regulate mitosis. In accordance, primary cilia play critical roles in sensory perception, and detection of neurotransmitters and hormones, regulation of cell division, development, and tissue regeneration [8–11]. Currently, little is known about the physiological and pathological function of neuronal and astrocytic primary cilia, despite their strong association with many human diseases including cognitive impairment [12], obesity [13], developmental disorders [14], and glioblastomas [15, 16].

Although neurons and astrocytes originate from the same precursors in early neurodevelopment, their primary cilia demonstrate a dichotomy in the mature brain [1]: their signaling pathways and molecular components, marker proteins [1, 17], ciliary nanoscale structure (communicated by Carolyn Ott from Janelia Research Campus in 2019), and functionality, as well as disease associations, are markedly diverged. In the adult brain, neuronal and astrocytic primary cilia are generally marked by type 3 adenylyl cyclase (AC3) [18] and ADP-ribosylation factor-like protein 13b (*Arl13b*), respectively [1]. AC3 represents a key enzyme mediating cAMP signaling in neuronal primary cilia throughout the mature brain [4]. Neurons are terminally differentiated and excitable cells that lose mitotic ability in maturity [19]. Neuronal primary cilia are relatively stable and lack de novo ciliogenesis in the adult brain. While no synaptic structures have been identified in neuronal primary cilia, many types of G protein-coupled receptors are detected in these tiny organelles [20]. Hence, neuronal cilia largely rely on metabotropic receptors and often AC3 to transduce a signal into the cell to regulate neuronal activity [4, 21, 22]. In contrast, astrocytes are non-excitable and generally do not intertwine with one another via chemical synapses. Moreover, they are not terminally differentiated cells and maintain proliferative capacity throughout life [23–25]. Since astrocytes can proliferate during reactive astrogliosis in the event of excitotoxic insults [23], astrocytic primary cilia are not static, but subject to dynamic change. Hence, it is not surprising that Arl13b, which regulates ciliary protein trafficking, the Sonic Hedgehog (Shh) pathway, and neural development, prevails over AC3 in astrocytic cilia as a protein marker [17, 26–29].

While neurons generally mediate electrical activity and neural communication in the mammalian brain [30], astrocytes are the most abundant cell type. Astrocytes support neurons by forming a part of the tripartite synapse [25], shuttling lactate for the supplementation of glucose [31], recycling neurotransmitters, maintaining homeostasis, and remodeling synapses [24]. In addition to providing neurotrophic factors, astrocytes play major roles in reducing and halting the progression of toxicity. In the event of harmful insults, astrocytes become reactive, and alter expression to protect healthy parts of the brain. Under these conditions, they change morphology by hypertrophying and extending processes towards the insults and sometimes proliferating to demarcate injury and form glial scarring [32]. These physical changes are accompanied by a heightened expression of GFAP in astrocyte processes [23]. Yet, it is unknown how astrocytic primary cilia change morphology and signaling components in the event of excitotoxic insults.

This study aimed to determine morphological changes and molecular alterations of neuronal and astrocytic primary cilia when the mouse brain is exposed to epileptic conditions and traumatic injury. We employed a double transgenic mouse strain, Arl13b-mCherry; Centrin2-GFP (named “Arl mice” henceforth), in which mCherry labels Arl13b-positive primary cilia, and GFP marks centrosomes via fusion with Centrin2 (a marker protein of centrosomes). This strain allows for direct visualization of Arl13b-positive primary cilia and centrosomes [33]. Serendipitously, we observed that the Arl mice exhibit a high incidence of spontaneously occurring seizures, which provided an excellent mouse model to examine the impact of spontaneous seizure activity on neuronal and astrocytic primary cilia. Additionally, we examined injured brain tissues and assessed the morphological changes of neuronal and astrocytic cilia after brain injury. Here we show that AC3-positive neuronal cilia shorten following reactive insults. Intriguingly, Arl13b-positive astrocytic cilia are not detectable, while Arl13b protein relocates to the cell body and has robust expression in the proximity of injured tissues.

## Methods and materials

### Mice

The Arl mice were introduced from the Jackson Laboratory (Stock No: 027967) and bred in house. The expression of Arl13b-mCherry and Centrin2-GFP was driven by CAG promoters [33–35]. This mouse strain has C57BL/6, FVB/N, and BALB/c mixed genetic background. Due to the strong activity of the CAG promoter, Arl13b was also found to be highly expressed in neurons in the hippocampus in adult Arl mice and prolong the length of neuronal primary cilia. Wild type FVB/N and C57BL/6 strains were purchased from the Jackson Laboratory and bred in house. Groups of mice were maintained on a 12 h light/dark cycle at 22 °C and had access to food and water ad libitum. Both male and female mice were used for all experiments to prevent sexual bias. All animal procedures were approved by the Institutional Animal Care and Use Committee of the University of New Hampshire and conducted in accordance with their guidelines.

### Observation of seizure activity

To determine the presence of the intermittent seizure activity, all Arl mice were observed individually for 30 s to 1 min in a new, clean cage after being removed from their home cage by their tail. Mice exhibiting seizure activity were marked with an animal marker, while mice without seizure expression were not labeled. Observations were repeated for 10 days over 2 consecutive weeks with additional observations occurring intermittently throughout the duration of this experiment. All mice were returned to their home cage after observation. A modified Racine scale was used to quantify seizure severity [36, 37]: 0, no change in behavior; 1, repetitive chewing; 2, head bobbing; 3, involuntary movement of forelimbs with tremors; 4, involuntary movement of all limbs with rearing and falling; and 5, hypertonia, loss of movement, and death.

### Primary astrocyte and neuronal cultures

P0-P3 pups were euthanized with sterile surgical scissors and isolated tissues were placed in ice cold HBSS (Corning, Reference: 21–040-CV). Meninges were removed fully while tissue remained immersed and chilled. Under sterile conditions, cortices were placed in prewarmed papain (Worthington-Biochem, Cat#: LS003127) in DMEM. DNAase was then added to mixture. Tissue was incubated at 37 °C for 15–20 min with inversion every 5 min. Tissue was then transferred to sterile conditions and washed three times in DMEM. Following this, tissue was dissociated into a single cell suspension by repeated pipetting with HBSS and settling of tissue. HBSS containing dissociated cells was centrifuged and pellet resuspended in astrocyte medium. To generate astrocyte cultures, cells were grown to confluence in flask for about 1 week and then split twice to produce pure astrocyte cultures. To produce neuronal cultures, media was removed after 2–4 h and cells were grown with neuronal media, following procedures as described previously [38]. Astrocytes and neurons were cultured in vitro for 7–10 days.

### Electroencephalogram/electromyogram (EEG/EMG) surgery, recordings, and analyses

Adult mice undergoing EEG/EMG surgeries were anesthetized with 1.5–3% isoflurane (Henry Schein, NDC 11695–6776-2) and secured in a stereotaxic device (Kopf Instruments). While under anesthesia, mice were prepared for surgery by hair removal, sterilization of skin with alcohol and 4% chlorhexidine (Phoenix, NDC 57319–612-09), and injections of 1 mg/kg lidocaine (Phoenix, NDC 57319–533-05) and 5 mg/kg carprofen (Putney, NDC 26637–521-02). Corneal drying was prevented by the application of sterile ophthalmic ointment (Solutions, Alta lube Ointment, X0020S6KF) prior to surgery. Once secured in the stereotaxic frame, the surface of the cranium was exposed with a single sagittal incision. The cranial surface was cleaned with saline and sterile gauze. Once dry, the EEG/EMG headmount (Pinnacle Technology Inc., Cat # 8201) was secured to the cranium with VetBond (3 M VetBond, 1469SB). Holes were drilled into the skull by hand with a 24-gauge sterile needle. Corners of the headmount were secured into the skull with Resin (Resin lab, SEC 12334GR) placed between the micro-screws (Pinnacle Technology Inc., Cat # 8209). After screws were tightened, the two probes attached to the back of the mount were placed directly into the spinotrapezius of the nuchal region of the mouse. Skin was then sutured around the base of the headmount. The mount and surrounding screws and wires were then stabilized with dental cement and allowed to dry. Mice recovered from surgery on a heating pad and were monitored until awake and active. Mice received carprofen administration for 1–3 days following surgery and were monitored for activity and weight fluctuations. To prevent sexual bias in our data, each phenotypic group had equal sexes of mice. Sirenia Seizure Pro software (Pinnacle Technology Inc. Version 1.8.3) was used to identify seizure activity. All events greater than 5 s were confirmed and interpreted through visual analysis.

### Cortical injury

Animals which underwent cranial surgery for device implantation were euthanized and their brain tissue was collected for immunohistochemistry. Following induction of sedation through isoflurane administration, animals were secured in a stereotaxic frame. A single incision was made along the sagittal suture and the cranium was exposed. Bone was cleaned with sterile cotton swabs and saline. Three holes were drilled into cranium with a sterile needle, and microscrews then secured an implant into place. Microscrews resulted in cortical damage and glial scarring not exceeding 2 mm. Microprobe implants were then attached to screws with dental acrylic for fixation. Mice were administered analgesics as necessary during their recovery prior to their use for other experiments.

### Transcardial perfusion and tissue fixation

Mice were deeply anesthetized with an intraperitoneal injection of ketamine (Ved Co, KetaVed, NDC 50989–161-06,)/xylazine (AKORN, NDC 59399–111-50). After confirmed assessment for lack of palpebral or tail pinch reflexes, they were transcardially perfused with phosphate buffered saline (PBS) followed by 4% paraformaldehyde (PFA) (Sigma-Aldrich, 252,549-1 L). In brief, a catheter was inserted into their left ventricle and the right atrium was punctured. PBS was administered at a rate of approximately 20 ml/min for 5 min, followed by PFA for roughly 7 min. Whole brains were extracted and then placed into 4% PFA overnight at 4 °C. The following day, brains were rinsed in PBS and placed into 30% sucrose in PBS for 48 h or until fully dehydrated. Tissue was then frozen directly on dry ice and stored at − 80 °C until use.

### Immunohistochemistry

After being embedded with tissue-tek optimal cutting temperature compound, brain tissue was sliced on a Leica CM3050 S cryostat to 30 μM sections at − 18 °C. For immunostaining, tissue was washed for 5–10 min 3 times in PBS while on an orbital shaker at room temperature. Tissue was then permeabilized in 0.5% PBST (PBS + Triton X-100) 3 times for 10 min during shaking. Tissue was then blocked for 2 h while shaking at room temperature in blocking solution (10% donkey serum (Sigma, D9663–10 ML), 2% bovine serum albumin (Sigma-Aldrich, A7906-100G), and 0.1 M glycine (Apex, 18–109) in 0.5% PBST). Tissue was then placed in new blocking buffer and incubated overnight while rocking at 4 °C with primary antibodies: anti-AC3 rabbit antibody (EnCor Biotechnology, AB2572219, 1:10,000 dilution), anti-Arl13b mouse (Neuromab, 75–287, 1:250), anti-GFAP rabbit (Dako, 2024-05-31, 1:750), and anti-GFAP mouse (Sigma, G3893-100UL, 1:500). After incubation, tissue was washed for 10 min 3 times in 0.5% PBST and then incubated with Alexa Fluor secondary antibodies 546 and 647 at 1:500 dilution. After incubation, tissue was washed once in 0.5% PBST for 10 min and then twice in PBS for 10 min. Floating sections were mounted on gelatin coated slides, allowed to moderately dry, and then fixed with DAPI mounting solution (Southern Biotech, 0100–20). Coverslips were secured with clear nail polish and allowed to dry fully before being stored at − 20 °C.

### Confocal microscopy and ImageJ analysis

Following immunofluorescence staining, tissue was imaged with a Nikon A1R HD25 confocal microscope, and tiled or non-tiled Z-stacks were taken for quantitative analysis. Fiji ImageJ was used to measure primary cilia, determine intensity of Arl13b with pixel analysis, and examine morphological differences of astrocytes and cilia within the hippocampus or injured cortical tissue. Cilia length was measured in the cornu ammonis 1 (CA1), cornu ammonis 3 (CA3), and dentate gyrus (DG) regions, and cortical injury sites of different strains and phenotypes of mice. Somatic differences characteristic of astrocyte reactivity were confirmed visually in the hippocampus or injured cortical tissue. Arl13b intensity was measured in 3–4500 μm lengths per region of the brain and then averaged per site.

### Data analysis

All length and intensity data were analyzed with GraphPad Prism and JMP statistical analysis software. Analyses included unpaired Student’s t-test, correlation analysis, Kolmogorov-Smirnov test, one-way ANOVA with Bonferroni correction, and density analysis. Significance was determined by a *p* value less than 0.05 (*), less than 0.01 (**), and less than 0.001 (***). Data in the graphs are presented as mean ± standard error of the mean.

## Results

### Arl mice have much longer astrocytic and neuronal primary cilia than wild type FVB/N and C57BL/6 mice

**Fig. 1:**
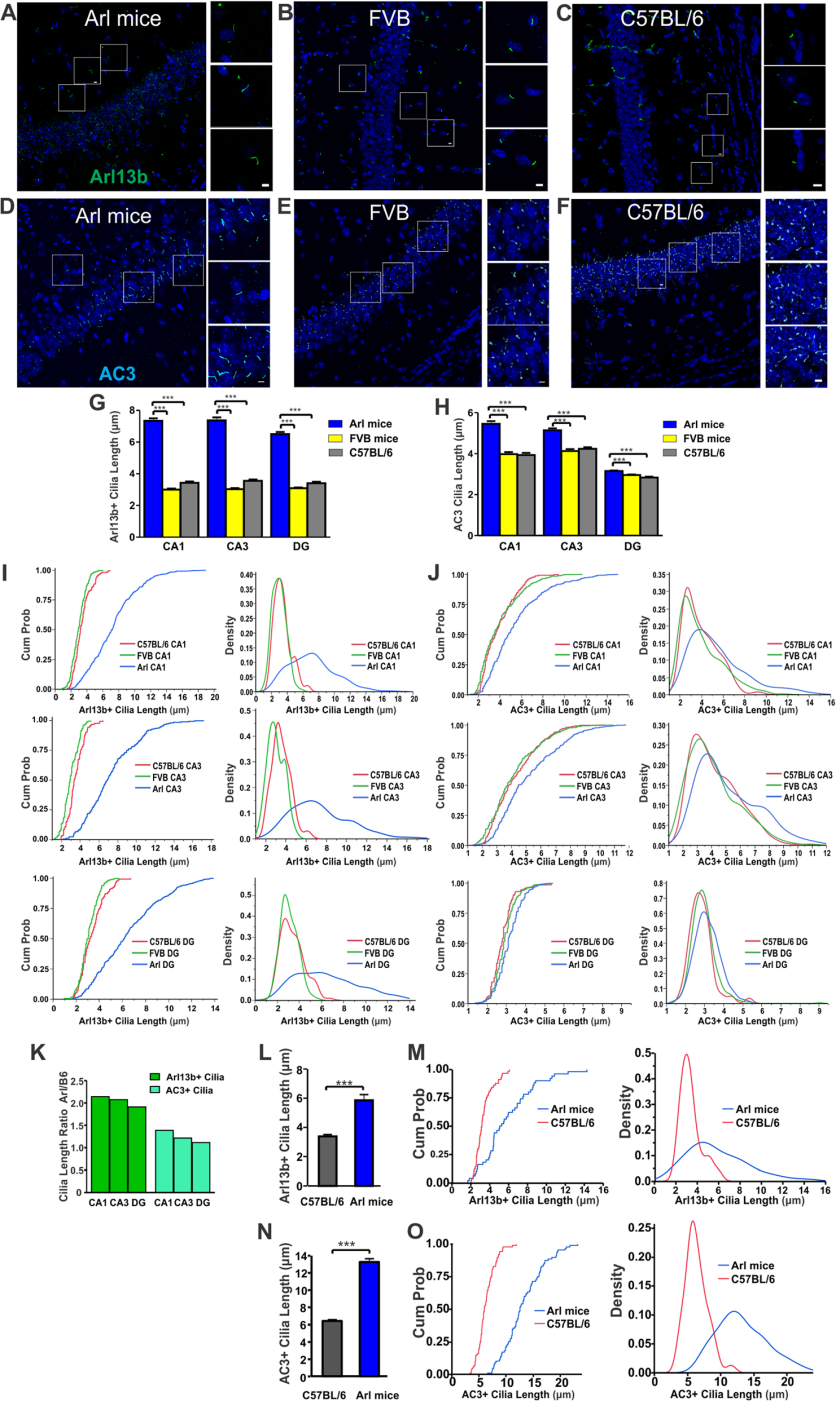
Arl13b-positive and AC3-positive primary cilia of Arl mice are longer than those of FVB/N and C57BL/6 wildtype mice. **a-c, g** Arl mice had longer Arl13b-positive primary cilia than control mice in the hippocampal CA1, CA3, and DG regions. **a** Arl mice, **b** FVB/N controls, **c** C57BL/6 controls. Representative images were taken from the CA1 region. Scale bar, 5 μm. **g** Comparison of Arl13b + cilia length in the hippocampal CA1, CA3, and DG of three mouse strains. Arl mice, *N* = 8; FVB/N mice, *N* = 5; C57BL/6, *N* = 4. Cilia number: Arl mice: 399, 230, and 435; FVB/N: 213, 146, and 254; C57BL/6: 144, 118, and 116. Data were compared with one-way ANOVA test with post hoc Tukey test. CA1: F(2, 753) =307, *p* < 0.001; CA3: F(2, 491) = 251, *p* < 0.001; DG: F(2, 802) = 241, *p* < 0.001. (**d**-**f**, **h**) Arl mice had longer AC3-positive primary cilia in three subregions in the hippocampus. **d** Arl mice, **e** FVB/N controls, **f** C57BL/6 controls. Scale bar, 5 μm. **h** Comparison of AC3+ primary cilia length in hippocampal CA1, CA3, and DG regions. Arl mice, *N* = 4; FVB/N, *N* = 5; C57BL/6, *N* = 3. Cilia number: Arl mice, 403, 471, and 527; FVB/N mice: 386, 343, and 555; C57BL/6: 202, 422, and 180. Data were analyzed with one-way ANOVA test with post hoc Tukey test. CA1: F(2, 988) = 58, *p* < 0.001; CA3: F(2, 1233) = 40, *p* < 0.001; DG: (2, 1259) = 21, *p* < 0.001. **i** CDFs and histogram density of cilia length show the distribution differences of Arl13b-positive cilia length in different hippocampal regions of the three mouse strains. **j** CDFs and histogram density of cilia length demonstrate the distribution differences of AC3-positive cilia length in different regions of three mouse strains. **k** Ratios of Arl13b- and AC3-positive cilia length in Arl mice relative to those of C57BL/6 mice. Cilia length ratios for Arl13b-positive primary cilia: CA1: 2.1; CA3: 2.1; and DG: 1.9; Cilia length ratios for AC3-positive primary cilia: CA1: 1.4; CA3: 1.2; and DG: 1.1. For (**a-k**), cilia length data were collected from 4 to 8 months old mice. **l-m** Primary cultures of astrocytes from Arl mice had much longer Arl13b-positive primary cilia than those from C57BL/6 mice. **l** Arl13b-positive cilia in primarily cultured astrocytes were significantly longer than those derived from C57BL/6 mice (***, *p* < 0.001, unpaired Student’s t-test). Data were collected from 6 Arl cultures, 5 C57BL/6 cultures. Cilia number: Arl mice, 50; C57BL/6 mice, 57. **m** CDFs and density comparisons demonstrate the differences in Arl13b-positive cilia length between Arl mice and C57BL/6 mice. **n** AC3-positive cilia in primarily cultured cortical neurons (~ 10 days in vitro) were significantly longer than those derived from C57BL/6 mice (***, *p* < 0.001, unpaired Student’s t-test). Data were collected from 6 Arl cultures, 6 C57BL/6 cultures. Cilia number: Arl mice, 87; C57BL/6 mice, 87. **o** CDFs and density comparisons demonstrate the differences in AC3-positive cilia length between Arl mice and C57BL/6 mice

Double transgenic Arl mice have become a useful mouse model to study primary cilia and centrosomes [33]. While this strain has been commonly used in developmental studies [33–35], little is known about their morphological features and cilia expression pattern in the mature mouse brain. To characterize cilia morphology of Arl mice, we first measured cilia length in the hippocampus. We chose FVB/N and C57BL/6 control mice for comparison, owing to the mixed genetic background of the Arl mice. The Arl mice have mCherry linked with Arl13b and do not require additional staining to detect Arl13b-postive primary cilia [33]. Thus, immunofluorescence staining for AC3 was completed in brain tissues of three strains of mice, while Arl13b immunostaining was only applied to FVB/N and C57BL/6 samples. We focused on the CA1, CA3, and DG regions in the hippocampus and measured cilia length in the three regions. Confocal imaging first revealed that Arl13b-positive cilia were longer in the CA3 region of Arl mice than that of both control strains (Fig. 1a-c). Cilia length quantification indicated that Arl mice have significantly longer Arl13b-positive cilia in the CA1, CA3, and DG regions than both control strains (Fig. 1g). Cumulative distribution frequency plots (CDFs) and histogram density comparisons also show a marked difference in cilia length in the three regions between Arl mice and the two control strains (Fig. 1i). Similar to Arl13b-positive cilia, AC3-positive neuronal primary cilia were also found to be longer in Arl mice than in FVB/N and C57BL/6 controls (Fig. 1d-f). Moreover, cilia length analysis revealed that Arl mice had significantly longer AC3-positive cilia in the hippocampal CA1, CA3, and DG regions than both control strains (Fig. 1h). CDFs and histogram density comparisons of AC3-positive cilia lengths also supported this conclusion (Fig. 1j). Interestingly, Arl13b overexpression affected Arl13b-positive cilia more intensely than AC3-positive cilia in adult mice (4–8 months old). Comparison of the ratios of Arl13b- and AC3-positive cilia length of Arl mice relative to that of C57BL/6 mice yielded a range of 1.9 to 2.1 for Arl13b-positive cilia, and 1.1 to 1.4 for AC3-positive cilia in the CA1, CA3, and DG regions, respectively (Fig. 1k). However, in primary astrocyte cultures derived from Arl mice, Arl13b-positive astrocytic cilia length was almost doubled compared to that derived from C57BL/6 mice (Fig. 1l&m). Primarily cultured neurons displayed a similar trend of lengthening in Arl mice when compared to controls, and the length of AC3-postive neuronal cilia were also doubled in neurons of derived from Arl mice (Fig. 1n&o). These data indicate that overexpression of Arl13b significantly increases the length of both astrocytic and neuronal primary cilia. These results are consistent with previous reports, showing that Arl13b regulates ciliary protein trafficking and promotes ciliogenesis [27, 29].

### Cilia length variation in the hippocampal CA1, CA3, and DG regions in three mouse strains

**Fig. 2:**
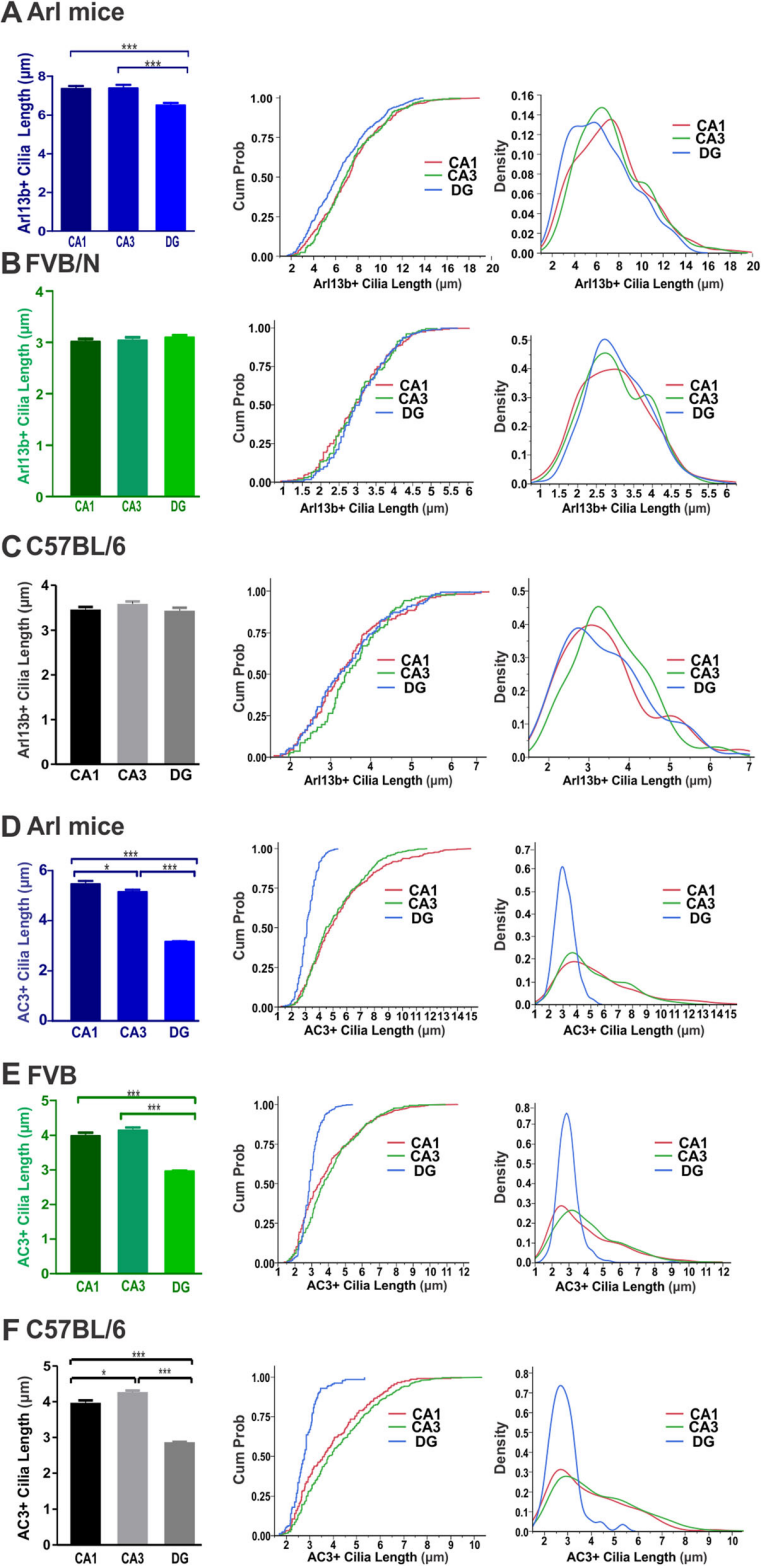
Regional cilia length variations in the hippocampal CA1, CA3 and DG in Arl mice, FVB/N and C57BL/6 mice. **a** Arl13b-positive cilia in hippocampal regions of Arl mice were significantly shorter cilia in the DG than in CA1 or CA3 (***, *p* < 0.001, unpaired Student’s t-test). *N* = 8 mice. Cilia number: 399, 230, and 435. **b** Arl13b-positive cilia of FVB/N mice had no significant differences in length among hippocampal CA1, CA3, and DG regions. *N* = 5 mice. Cilia number: 213, 146, and 254. **c** Arl13b-positive cilia of C57BL/6 mice had no significant differences in length among the hippocampal CA1, CA3, and DG regions. *N* = 4 mice. Cilia number: 144, 118, and 116. **d** AC3-positive cilia in the hippocampal DG region of Arl mice were significantly (***, *p* < 0.001) shorter than in the CA1 and CA3 regions, and significantly shorter in the CA3 than in the CA1 (*, *p* < 0.05). *N* = 4 mice. Cilia number: 403, 471, and 527. **e** AC3-positive cilia in hippocampal DG regions of FVB/N control mice were significantly (***, *p* < 0.001) shorter than in the CA1 and CA3 regions. *N* = 5 mice. Cilia number: 386, 343, and 555. **f** AC3-positive cilia in hippocampal DG regions of C57BL/6 mice were significantly (***, *p* < 0.001) shorter than in the CA1 and CA3 regions. *N* = 4. Cilia number: 202, 422, and 180. Data were analyzed with one-way ANOVA with post hoc Tukey test. Arl13b: Arl strain: F(2, 1061) = 11.05, *p* < 0.001; FVB: F(2, 610) = 0.5850, *p* = 0.4632; C57BL/6: F(F(2, 3775) = 0.7700, *p* = 0.4632; AC3: Arl Strain: F(2, 1398) = 213.6, *p* < 0.001; FVB: F(2, 1281) = 93.82, *p* < 0.001; C57BL/6: F(2, 801) = 62.96, *p* < 0.001

To examine the length variation of Arl13b-positive and AC3-positive cilia in different regions of the hippocampus among different mouse strains, we compared the cilia lengths in the CA1, CA3, and DG regions. Arl13b-positive and AC3-positive cilia of Arl mice are significantly shorter in the DG than in the CA1 and CA3 regions (Fig. 2a&d). Additionally, AC3-positive cilia in CA3 were moderately shorter than those in the CA1 in Arl mice (Fig. 2d). However, there was no significant regional difference in Arl13b-positive cilia length in FVB/N or C57BL/6 mice (Fig. 2b-c). Moreover, AC3-positive cilia were found to be significantly shorter in the DG than in the CA1 and CA3 regions in FVB/N and C57BL/6 mice, and moderately longer in the CA3 when compared to the CA1 in C57BL/6 mice (Fig. 2e-f). These results on regional cilia length differences are in line with another study, reporting that AC3-positive neuronal cilia are shortest in the DG region in the hippocampus [39].

### Centrin2-GFP centrosome protein does not consistently localize at the base of primary cilia in the adult brain

**Fig. 3:**
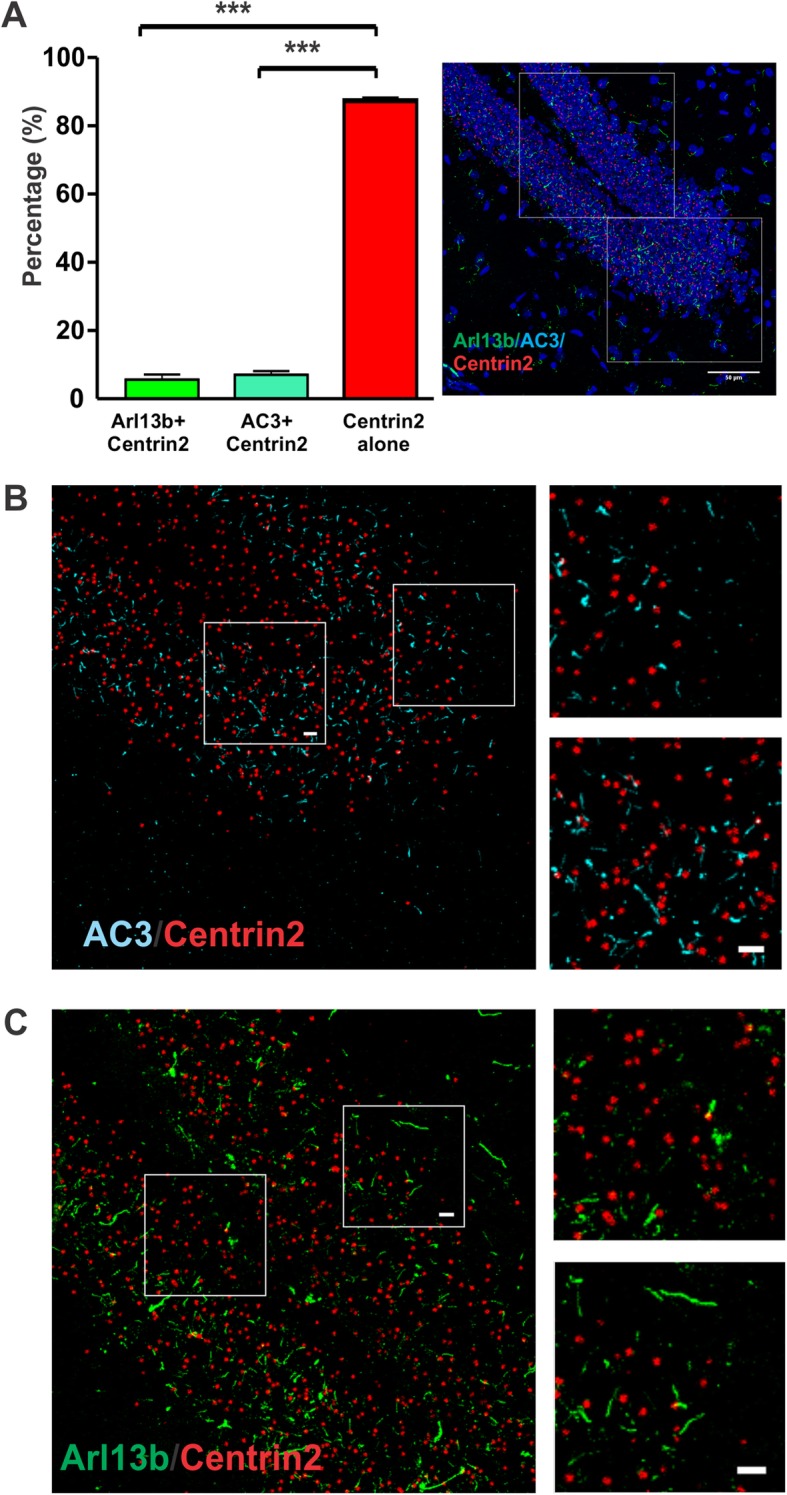
Centrin2-GFP is not consistently localized at the base of primary cilia. **a** The percentage of co-expression of Centrin2 with known cilia markers (AC3 or Arl13b) had significant difference from Centrin2 alone (***, *p* < 0.001, unpaired Student’s t-test). *N* = 5 Arl mice. Cilia number: Arl13b-positive, 48; AC3-positive: 46. Centrin2: 446. **b-c** Zoom-in images from the image of A. Centrin2 was not always present at the base of Arl13b-positive cilia or AC3-postive cilia. Centrin2 imaging density over 50 μm2 regions per mouse was calculated. Green: Arl13b, Cyan: AC3, Red: Centrin2. Scale bar: 5 μm

The mother centriole forms the base of the primary cilium, and comprises the centrosome with the daughter centriole during interphase [1, 40]. It has been widely thought that Centrin2 is a marker protein of the centrosome, and the Centrin2-GFP of Arl mice has been used to mark the centrosome in multiple developmental studies [33, 41–44]. Nevertheless, it has not been assessed if Centrin2-GFP can mark the centrosomes of all primary cilia in the mature brain. We determined the co-expression percentage of Centrin2 with Arl13b and AC3 (Fig. 3). Centrin2 was expected to be found at the base of neuronal primary cilia. However, the image quantification of percentages of co-expression shows a significantly higher amount of non-ciliated Centrin2 than Arl13b-Centrin2 or AC3-Centrin2 co-expression (Fig. 3a-c). These data suggest that Centrin2-GFP does not always localize to the base of neuronal primary cilia or astrocytic cilia, and thus cannot be always used as a protein marker to mark the location of centrosomes of primary cilia in the hippocampus of Arl mice.

### Arl mice exhibit a high incidence of spontaneous seizure

**Fig. 4:**
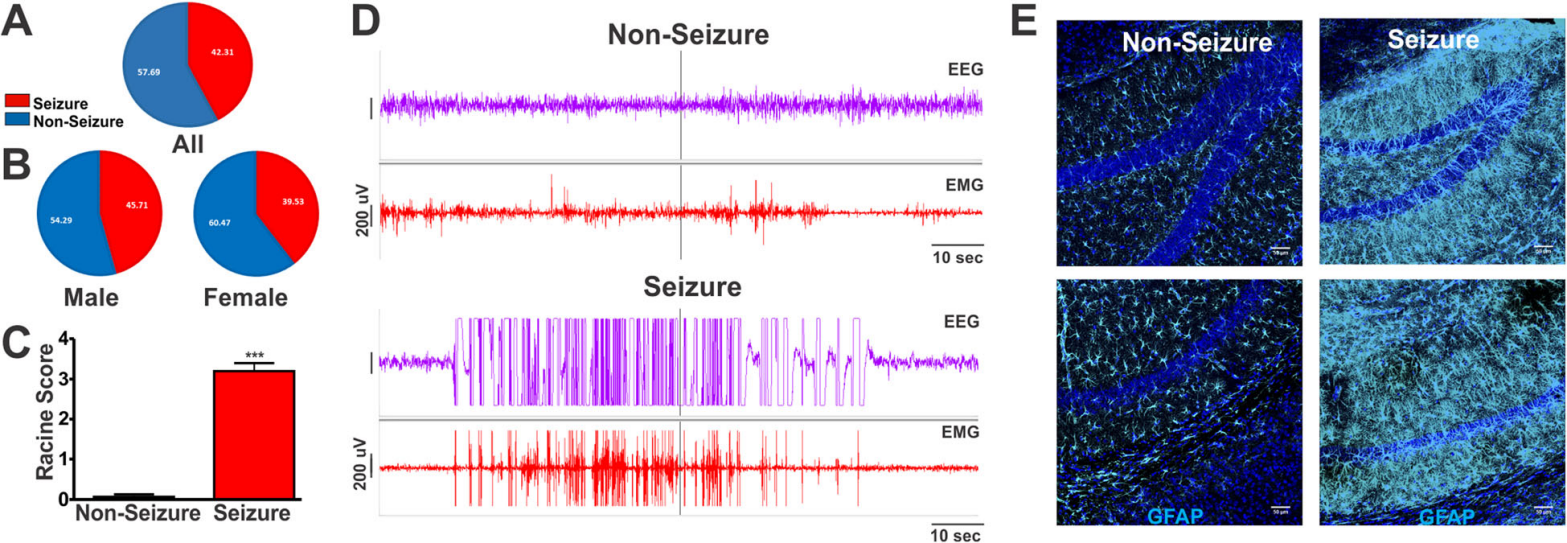
Arl mice exhibit a high incidence of spontaneous seizure activity. **a** Naturally occurring seizures occur in a large proportion of Arl mice. Blue: non-seizure; red: seizure. *N* = 78. **b** Both male and female Arl mice had seizure activity. Males had 16 mice out of 33 total exhibiting seizures. Females had 17 seizure mice out of 43 total. **c** Arl seizure mice exhibit a high Racine score on average. 15 non-seizure mice were compared with 10 seizure mice. ***, *p* < 0.001, unpaired Student’s t-test. **d** EEG/EMG recordings confirmed the occurrence of seizures among Arl mice. Epileptic waveform of EEG/EMG recording verified seizure activity in a mouse grading high on Racine score (bottom), compared to non-seizure Arl mice which lacked high-amplitude EEG/EMG waves (top). **e** Immunofluorescence staining of non-seizure and seizure brain tissue shows drastically elevated GFAP expression in tissues from seizure mice, indicative of astrocyte reactivity. Top, DG; bottom, CA1 region. Scale bar, 50 μm


Likely due to the hybrid background strains of the Arl mice, we also observed spontaneous seizure activity in Arl mice (Fig. 4a). This epileptic activity is short-lived and rarely correlated with sudden death or chronic incapacity in mice. To determine the prevalence of this phenotype in our cohort, all adult mice were first observed daily for 10 days over 2 consecutive weeks. During observations, we removed mice from their home cage by a tail lift and then placed them individually in a new, clean cage for 30 s to 1 min. These standard husbandry procedures were usually enough to initiate seizure activity in Arl mice. This epileptic activity manifested in mice more as they aged and was non-existent in mice under 2 months old. Seizure activity occurred among 42% of a population of 78 Arl mice (4–8 months). Epileptic activity often lasted from 5 to 15 s, and after roughly 10 s following the event, the mouse would return to normal activity. There was no significant sexual bias in seizure prevalence of the Arl mice population (Fig. 4b). To evaluate the degree of seizure activity, we used a modified Racine Scale to quantify seizure severity (Fig. 4c & Supplemental Video 1). We found that approximately all mice exhibiting seizures scored between 3 and 4 on a modified Racine Scale (Fig. 4c & Supplemental Video 1). We rarely found mild seizures occurring in this population and did not experience any seizure related deaths or disruptions of fertility. To verify that the spontaneously occurring spastic patterns in the Arl mice were caused by epileptic activity in the brain and not by skeletal muscle spasms, we performed EEG/EMG recordings on 8 Arl mice as well as C57BL/6 control mice. Of the 8 Arl mice used in this experiment, half were known to exhibit seizures and half had no known history of seizure activity. Mice were recorded with EEG/EMG equipment for 48 h. We evaluated EEG/EMG recordings with Sirenia Seizure Pro software and manual inspection of the EEG/EMG waves to detect epileptic events. 17 seizure events occurred during this 48-h period, distinguished by a high amplitude of EEG waves lasting for a time period no less than 5 s (Fig. 4d). Of the 17 seizures (11 from females and 6 from males) recorded, erratic epileptic activity lasted from a range of 16 to 126 s, with an average time of 52 s. In contrast, we did not observe any epileptic activity in C57BL6 mice.

**Additional file 1: Video S1.** Arl mice exhibit spontaneous seizure. Two adult Arl mice displayed seizure activity after cage change.


Seizures are known to cause astrocyte reactivity, a condition commonly correlated with many neurological diseases [45, 46]. Next, we sought to determine if the naturally occurring seizures resulted in astrocyte reactivity. If so, brains of Arl seizure mice would maintain a high level of GFAP intensity and hypertrophied astrocytes with additional long processes. We chose to examine these characteristics, because GFAP is a commonly accepted astrocyte marker that increases expression under reactive conditions, and morphological changes in these cells are characteristic in the development of reactivity [47–50]. Figure 4e shows that Arl mice with a known history of seizures were found to display heightened intensity of GFAP and additional processes of GFAP positive astrocytes when compared to controls. These morphological changes and alterations in GFAP expression are consistent with the development of astrocyte reactivity in the brain [23, 48–50]. These data indicate that Arl mice exhibit spontaneous seizure activity, which could be used as a model to study the effect of seizure-induced astrocyte reactivity on primary cilia.

### Naturally occurring seizures reduce the length of astrocyte and neuronal primary cilia in the hippocampus

**Fig. 5:**
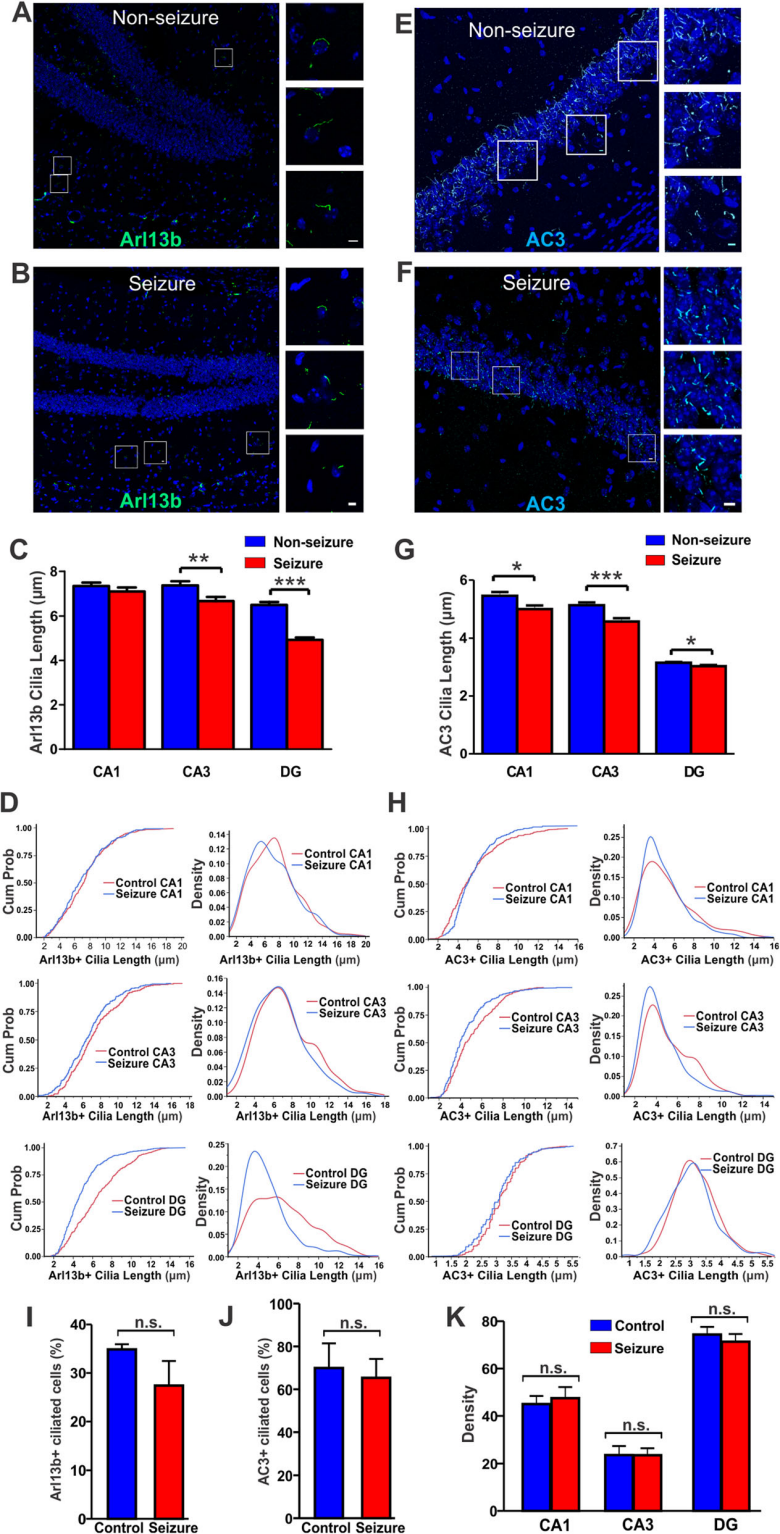
Naturally occurring seizures reduce the length of AC3-positive and Arl13b-positive primary cilia in the hippocampus. **a-b** Arl13b-positive cilia in the hippocampal CA1 and DG regions of Arl seizure mice (**b**) were shorter than those of non-seizure Arl control mice (**a**). Scale bar: 5 μm. **c** The length of Arl13b-positive cilia in the CA1 and DG regions, but not in the CA3 region, was significantly shorter in Arl seizure mice than in Arl non-seizure mice (***, *p* < 0.001, unpaired Student’s t-test). Arl non-seizure controls, *N* = 4; Arl seizure mice, *N* = 5. Cilia number: Non-seizure mice: 160, 130, and 253; Seizure mice: 180, 131, and 183. **d** CDFs and histogram density comparison present the length of Arl13b-positive cilia in the CA1, CA3, and DG regions. **e-f** AC3-positive cilia in the CA3 region of Arl non-seizure control mice (**e**) and Arl seizure mice (**f**). Scale: 5 μm. **g** Cilia length comparison revealed the shortening of AC3-positive cilia in three hippocampal regions of seizure mice (CA1: *, *p* < 0.05, CA3: ***, *p* < 0.001, DG: *, *p* < 0.05, unpaired Student’s t-test). Non-seizure controls, *N* = 4; seizure mice, *N* = 5 animals. Cilia number: non-seizure mice: 403, 471, and 527; seizure mice: 374, 294, and 375. **h** CDFs and histogram density show AC3-positive cilia length in the hippocampal CA1, CA3, and DG regions. **i** Seizure does not significantly affect the percent of Arl13b-positive cilia in the DG. Data collected from 3 non-seizure and 3 seizure Arl mice. Cell number: control: 185; seizure: 196. Cilia number: control: 64; seizure: 51. n.s. not significant, with unpaired Student’s t-test. **j** Seizure does not significantly affect the percent of AC3-positive cilia in the DG. Data collected from 3 non-seizure and 3 seizure Arl mice. Cell number: control: 292; seizure: 414. Cilia number: control: 221; seizure: 282. n.s. not significant, with unpaired Student’s t-test. **k** Centrin2-GFP expression in hippocampal regions in Arl seizure mice had no significant difference with that in Arl non-seizure mice. Centrin2 imaging density over 50 μm^2^ regions per mouse was calculated. Data were collected from 3 non-seizure and 3 seizure Arl mice. *p* = 0.6, 0.9, and 0.5 respectively with unpaired Student’s t-test

AC3 is mostly enriched in neuronal primary cilia, while Arl13b is widely expressed in astrocytic primary cilia in the adult mouse brain [1, 17]. To date, it is not clear how spontaneous seizures affect Arl13b-positive astrocytic cilia and AC3-positive neuronal cilia. As Arl mice exhibiting seizures conferred an excellent model of excitotoxicity, we sought to examine if seizures cause any morphological changes in Arl13b- and AC3-positive primary cilia. We stained and analyzed brain tissue of Arl mice with a known history of seizures and Arl mice with no known history of seizure activity. Our immunostaining results showed that Arl seizure mice had shorter Arl13b-positive cilia in the DG than those of Arl mice with no known history of seizures (Fig. 5a-b). Quantification of cilia length showed significantly shorter cilia in the CA3 and the DG, but not in the CA1 (Fig. 5c-d). Moreover, analyses of AC3-positive cilia of Arl seizure mice showed a marked reduction of AC3-positive cilia length in the CA3 region and a moderate reduction in length in the CA1 and the DG compared to those of non-seizure mice (Fig. 5e-h). Nevertheless, the ciliation frequency of Arl13b- and AC3-positive cilia was not significantly changed in the DG region by seizure (Fig. 5i&j). Additionally, the presence of seizures did not alter Centrin2-GFP expression in the three regions of the hippocampus of Arl mice (Fig. 5k). Thus, spontaneous seizures reduce the length of both neuronal and astrocytic primary cilia in regions of the hippocampus.

### Elevated Arl13b expression proximal to injured tissue

**Fig. 6:**
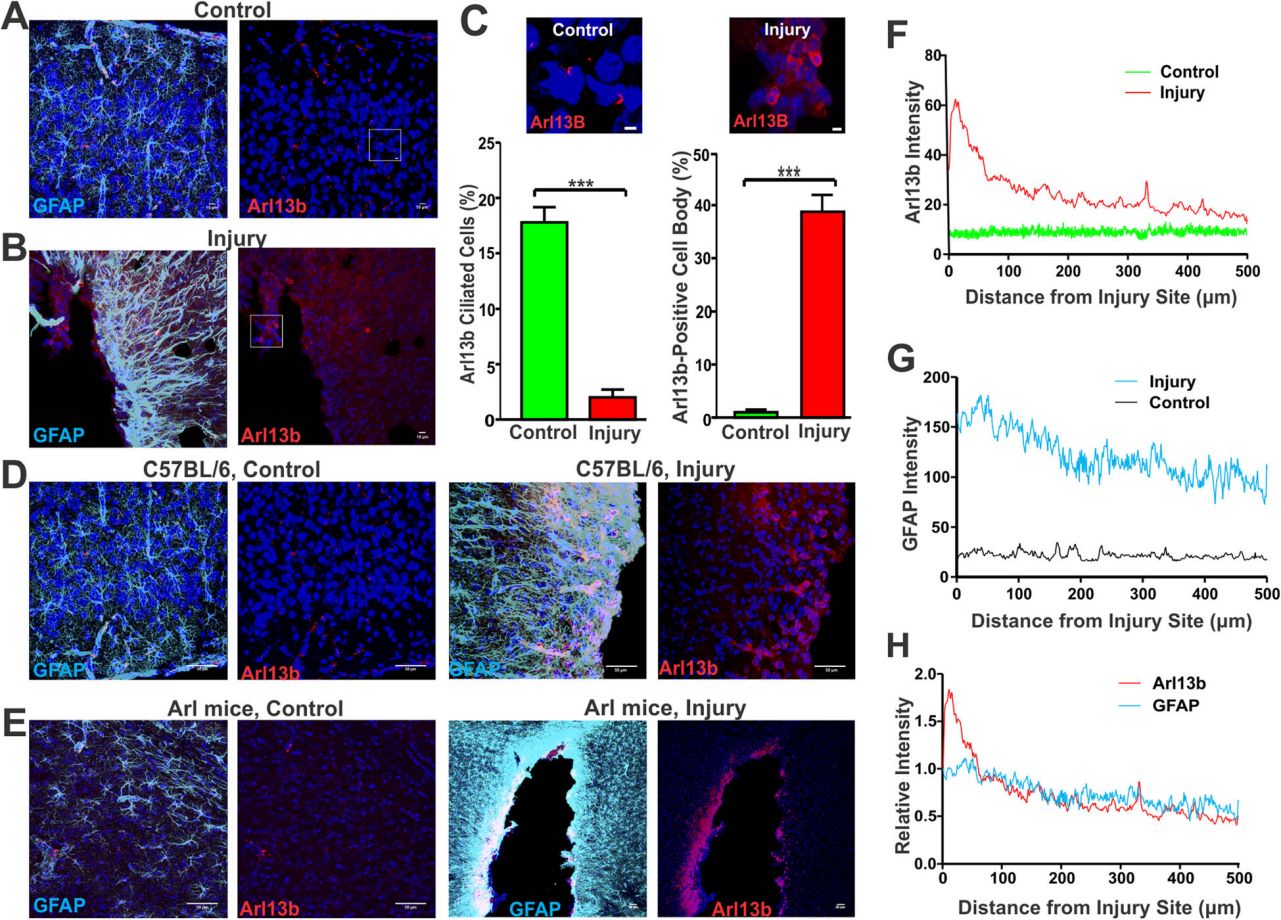
Absence of Arl13b-positive astrocytic primary cilia but robust Arl13b expression in tissues proximal to injured sites. **a-b** Increased Arl13b expression proximal to an injury site (**b**) compared to a non-injury site (A). Scale: 5 μm. Note that Arl13b-positive primary cilia were detected in non-injured tissues (**a**), but undetectable in injured tissues (**b**). **c** The percentage of ciliated cells near injury sites decreased dramatically compared to control tissues (left). ***, *p* < 0.001, unpaired student’s t-test. Injured tissues: total cell number: 665, ciliated cell N: 13; non-injured tissues: total cell number: 265, ciliated cell number: 47. Injured tissues had significantly increased percentage of Arl13b-positive cells compared to that of non-injury tissues (right). ***, *p* < 0.001, unpaired Student’s t-test, *N* = 3. Injury site cell *N* = 806; non-injury site cell number 600. Two Arl13b images from injured and non-injured sites are shown in the left. Non-injury sites were from the opposite hemisphere of the injury site of same mouse. **d** Increased Arl13b expression near injured sites in the cortex of C57BL/6 mice. GFAP (cyan) and Arl13b (red) were stained with their antibodies, respectively. Left, non-injured site; right, injured site. **e** Increased Arl13b expression near injured sites in Arl mice. Red: Arl13b-mCherry; Cyan: immunofluorescence staining using anti-GFAP antibody. **f** Distinct Arl13b expression density in injured and non-injured tissues. Arl13b had the highest expression near to the injury site. *N* = 5. **g** GFAP expression was strongly increased near to injured tissues compared to non-injured tissues. **h** Correlation of relative expression density of GFAP and Arl13b over a range of 500 μm distance from the injured site. ***, *p* < 0.001 by correlation analysis

To further evaluate the changes of primary cilia under reactive conditions, we employed a model of traumatic brain injury, which causes the development of astrocyte reactivity, the generation of glial scarring [51], and is often used in brain injury research [52, 53]. We used a cortical injury model and assessed tissue around injury sites. Surprisingly, we failed to detect the presence of Arl13b-positive cilia proximal to the damaged tissue. Interestingly, although Arl13b-positive cilia were undetectable, Arl13b fluorescence intensity drastically increased in cell bodies in proximity to injured tissues (Fig. 6a-c). The percentage of Arl13b-positive cells proximal to the injury site was significantly higher than that in control tissue of the contralateral hemisphere of the same brain (Fig. 6c). To exclude the possibility that the increase of Arl13b is simply caused by an immunostaining background, we compared Arl mice with no known history of seizures with a cortical injury with C57BL/6 control mice with a cortical injury. Both mCherry signal and immunofluorescence staining using Arl13b antibody yielded similar results: Arl13b-positive cilia were undetectable, while the Arl13b expression shifted to the cell body and was strongly elevated near injury sites (Fig. 6d-e). We employed ImageJ to quantify Arl13b expression intensity over a range of 500 μm from the injury site. Arl13b expression intensity was found to be substantially higher close to the damaged tissue and decrease with distance from the injured site (Fig. 6f). In contrast, Arl13b intensity in control tissue (the opposite hemispheres of the same animals) remained consistent and at low levels (Fig. 6f). Similarly, GFAP exhibited increased expression near injured tissues, indicative of reactive astrogliosis in the glial scar (Fig. 6g). Interestingly, the relative expression density of Arl13b correlated with that of GFAP expression in the proximity of injured tissues (Fig. 6h). The relocation of Arl13b expression to the cell body suggests that Arl13b may regulate cell division or tissue repair in injured tissues.

### Brain injury shortens AC3-positive primary cilia

**Fig. 7:**
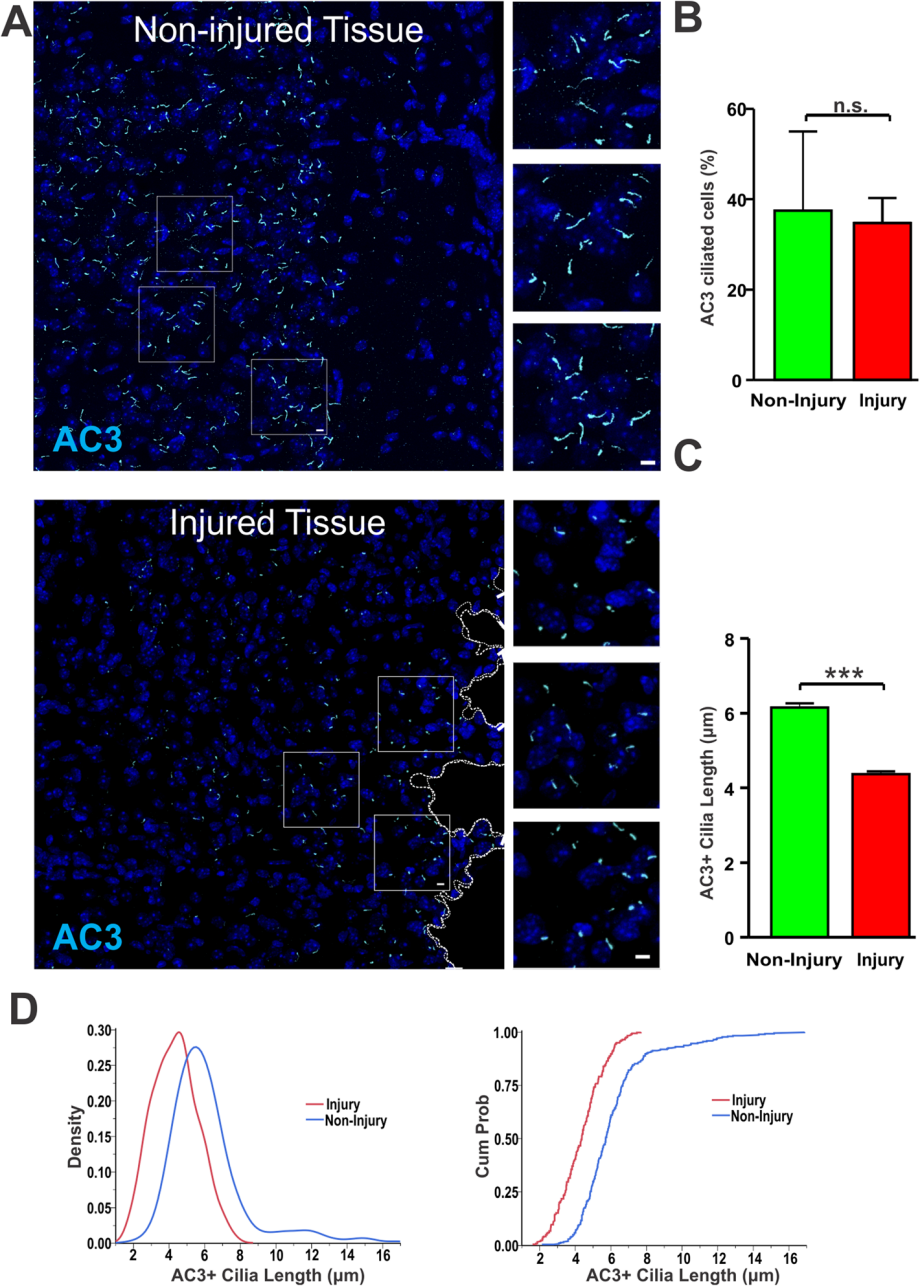
Tissue injury decreases the length of AC3-positive primary cilia. **a** AC3-positive cilia were shortened close to injured tissues. Scale bar: 5 μm. Top, non-injured tissues; bottom, injured tissues. **b** The frequency of AC3-positive cilia was not changed between injured and non-injured tissues (n.s. not significant, unpaired Student’s t-test). **c** Comparison of AC3-postive cilia lengths reveals significant decrease in cilia length proximal to injury sites compared to injured tissues (*** *p* < 0.001, unpaired Student’s t-test). *N* = 5, C57BL/6 mice. Non-injured sites were from opposite hemisphere of the injury site of the same animals. Cilia number: non-injured sites: 271; injured sites: 326. **d** CDFs and density comparison indicate the shortening of AC3-positive cilia length near to injury sites

Next, we examined AC3-positive primary cilia near injury sites to determine if effects were consistent with the shortening effect of seizures. We found that, unlike Arl13b-positive cilia, AC3-positive cilia were discernable among neurons close to the injury sites, excluding the immediate glial scarring (Fig. 7a). There was no obvious shift in the localization of AC3 to the cell body and all visible AC3-positive cilia maintained proper shape (Fig. 7a). The frequency of AC3-positive cilia in injured tissues was not significantly changed compared to non-injured tissues (Fig. 7b). However, the length of AC3-positive cilia was markedly shorter near damaged tissues than those in non-injured tissues (Fig. 7c). CDFs and density comparisons also confirmed the reduction of cilia length (Fig. 7d). These data suggest that although brain injury does not cause dismantling of neuronal cilia, it does decrease their length. These results, together with the seizure-induced shortening on neuronal cilia, suggest an adaptive morphological change under reactive conditions to reduce ciliary signals being sent to neurons.

## Discussion

Arl mice contain fluorescent proteins that label Arl13b-positive primary cilia and Centrin2-positive centrosomes [33]. This strain has been used to investigate the roles of primary cilia in development [33–35]. We first found that overexpression of Arl13b significantly elongates both astrocyte and neuronal primary cilia in the hippocampus. The longer cilia suggest possible gain-of-function features, which may correlate with augmented function. We also show that a high percentage of Arl mice exhibit spontaneous seizures. This phenotype could be partly due to genetic background of the Arl strain, which contains a hybrid cross with the FVB/N strain. Populations of FVB/N mice are known have higher seizure incidence than other strains. Seizure activity in FVB/N mice has been associated with astrocyte reactivity and neuronal death [54]. Similar to our cohort of Arl mice, seizures are known to emerge in FVB/N mice at 2–16 months of age as a result of mild to moderate stimuli [55]. However, the seizure incidence in our population of Arl mice (42%) is much higher than what has been reported in FVB/N mice (17–20%) [56, 57], raising the possibility that this difference may be caused by the transgenic overexpression of Arl13b and increased length of neuronal primary cilia in the brain. Elongated neuronal primary cilia could potentially elevate basal neuronal activity, consequently increasing the incidence of seizures. Thus, we postulate that the epileptic activity among Arl mice is partly due to the partial FVB background and elongated neuronal primary cilia may increase the seizure frequency. However, long-term spontaneous seizure in mice may in turn lead to the shortening of neuronal primary cilia, which may result from ciliary adaptive change under excitotoxicity.

Neurons are terminally differentiated cells and lose the ability to divide at maturity. Hence, it is not surprising to observe that neuronal primary cilia are relatively stable and the ciliation frequency does not change under seizure (Fig. 5) and traumatic brain injury (Fig. 7). Nevertheless, neuronal primary cilia become shorter in all regions of the hippocampus in Arl mice under spontaneous seizures. The shortening of AC3-positive cilia perhaps reflects adaptive changes of neuronal primary cilia in response to excitotoxicity insults. It has been reported that a disruption of radial patterning of neuronal primary cilia, as well as the absence of change in glial cilia in the hippocampus accompany a pilocarpine-induced seizure model [58]. In our study, although neuronal primary cilia were found to become shorter, we cannot verify the disruption of radial patterning of neuronal primary cilia upon seizure. The different results may be due to the spontaneous and long-term seizure occurrence versus brief seizure occurrence induced by a convulsant. Additionally, the significantly longer Arl13b-positive primary cilia in the Arl mice may also affect the increased seizure occurrence, due to alterations in signaling or possibly variations in astrocyte function decreasing their ability to recycle synaptic neurotransmitters. It is possible that the ciliary shortening in AC3-positive primary cilia may be due to alterations in ciliary protein receptors. Yet, the exact function of neuronal primary cilia is not well appreciated, and further research is required to understand how neuronal ciliary signaling regulates neuronal excitability and epileptic conditions.

Astrocytes are the most abundant cells in the mammalian brain and fulfill a variety of physiological functions, including providing trophic support neurons, and recycling neurotransmitters, maintaining homeostasis, and remodeling synapses [59–62]. Unlike neurons, astrocytes maintain the capacity of cell division, and new astrocytes are continuously generated in many regions during postnatal development [63]. Astrocytes become reactive in response to reactive insults, including epilepsy [64, 65] and traumatic brain injury [66]. Under these neuropathological events, they proliferate upon the initiation of reactivity [62, 67–69]. In this study, we first examined the effects of spontaneously occurring seizures on primary cilia. We found that there was a shortening of Arl13b-positive primary cilia in regions of the hippocampus. To further determine the morphological change of primary cilia during astrocyte reactivity, we employed a cortical injury model and examined primary cilia close to the injury sites. Arl13b-positive primary cilia were not detectable near injured tissues. Surprisingly, Arl13b relocated to the cell body and its protein expression is drastically intensified in the proximity of injured tissues (Fig. 6). Limited by available antibodies, we were unable to definitively prove if astrocytic cilia were ablated near the injury site or if the Arl13B expression merely shifted. The increased expression of Arl13b is correlated with elevated GFAP expression, suggesting a role of Arl13b in astrocyte proliferation and tissue repair.

Arl13b is a member of the Ras family of GTPases [27]. Mutations in Arl13b are known to result in defective cilia formation leading to Joubert syndrome, a ciliopathy characterized by neurological abnormalities and cognitive delay [70]. Arl13b associates with the ciliary membrane through palmitoylation and is required for trafficking of proteins within primary cilia [71]. Moreover, Arl13b is also known to regulate the trafficking of Shh signaling components to primary cilia, elements known to have a role in reparations following brain injury [28]. Relevantly, disruption of Arl13b inhibits Shh signaling over-activation and suppresses medulloblastoma formation [72]. It is well recognized that the Shh pathway mediates a developmental signal pathway vital to proper neural tube formation and body patterning [73, 74]. Defects in Shh can result in severe physiological abnormalities [75–77]. Mutations of Arl13b correlate with deformed primary cilia and abrogated Shh modulation [27] Shh induction increases following the acute phase after brain injury, thereby enhancing the proliferation of astrocytes under reactive conditions [78]. Given the strong association between Arl13b and Shh signaling, Arl13b translocation from the primary cilium to the cell body may regulate Shh signal transduction for tissue regeneration in the event of brain injury.

In summary, our findings validate the Arl strain as a useful mouse model to study primary cilia, which could be used as a gain-of-function tool to study the roles of both astrocytic and neuronal primary cilia. We also present the evidence of spontaneous seizure occurrence among Arl mice and unravel the effects of spontaneous seizures on astrocyte and neuronal primary cilia. Both types of primary cilia in the hippocampus are found to be shortened in reactivity induced by spontaneous seizure conditions. We further reveal that traumatic brain injury not only shortens neuronal primary cilia, but also highly elevates Arl13b expression proximal to the injury sites, implicating Arl13b in the regulation of astrocyte reactivity and tissue regeneration. This work suggests that the Arl13b-mediated signaling may represent potential therapeutic targets for tissue repair [8]. However, further studies are warranted to elucidate the molecular mechanisms of astrocytic primary cilia and Arl13b-mediated signaling in tissue regeneration following brain injury.

## Data Availability

The data generated by this work will be made accessible to the public.
